# Improving the Heat Resistance and Flame Retardancy of Epoxy Resin Composites by Novel Multifunctional Cyclophosphazene Derivatives

**DOI:** 10.3390/polym15010059

**Published:** 2022-12-23

**Authors:** Wangxi Fan, Zefang Li, Qin Liao, Lintong Zhang, Longjie Kong, Zhou Yang, Meng Xiang

**Affiliations:** 1 School of Chemistry and Chemical Engineering, Jiangsu University of Technology, Changzhou 213001, China; 2 School of Computer Engineering, Jiangsu University of Technology, Changzhou 213001, China; 3 School of Materials Science and Engineering, Changzhou University, Changzhou 213164, China

**Keywords:** epoxy resins, heat resistance, cyclophosphazene, flame retardancy

## Abstract

A novel multiple-ring molecule containing P and N, called HCCP-SA, was successfully prepared by the nucleophilic substitution reaction of salicylamide (SA) and hexachlorocyclotriphosphazene (HCCP). Particularly, HCCP-SA possessed the dual functions of heat resistance and flame retardancy. The molecular structure of HCCP-SA was identified by Fourier transform infrared spectroscopy and nuclear magnetic resonance spectroscopy. HCCP-SA was bonded into the molecular chain of epoxy resin by the ring-opening curing reaction of epoxy resin, aiming to form a heat-resistant and flame-retardant composite (E-HS-*x*). In particular, the best-prepared E-HS-*x* composite with a 20 phr content of HCCP-SA (E-HS-20) presented excellent thermal stability, with an initial decomposition temperature of 267.94 °C and a max weight loss speed of only 0.95 mg·min^−1^. Moreover, E-HS-20 exhibited remarkable flame retardancy with a limiting oxygen index value of 27.1% and a V-2 rating in the UL94 flame retardancy test. The best-prepared E-HS-20 composite would be a suitable and potential candidate for heat-resistant and flame-retardant polymer materials.

## 1. Introduction

As a kind of commercial thermosetting material, epoxy resins (EP) possess excellent mechanical strength, thermal stability, adhesion and electrical insulation [1,2,3]; therefore, they are widely used in the field of electronic information technology, such as packaging materials for electronic components, anti-corrosion coatings, etc. With the rapid development of semiconductor technology and integrated circuit integration, the heat generation of electronic components has increased dramatically in recent years. Accordingly, advanced high-density installation technology (especially large and super large-scale integrated circuits) and thin packaging technology urgently require that the heat resistance and flame resistance of epoxy resin be simultaneously improved, in order to meet the industrial demand in all walks of life.

The improvement of the heat resistance of electronic packaging materials can usually be achieved by increasing the crosslinking degree of epoxy resin [4,5,6]; however, the brittleness of epoxy resin will increase if the crosslinking degree is too high. A large number of investigations have shown that the introduction of additives containing phenyl and naphthyl groups in epoxy resin can also improve the heat resistance [7,8,9]; however, it is a challenge to build a new multi-ring structure in epoxy resin to improve its heat resistance.

Two main methods to obtain epoxy resin with high flame retardancy are to add flame retardants and/or prepare inherent flame retardants by embedding flame-retardant groups in the molecular chain of epoxy resin. The addition of flame retardants usually shows some limitations. For example, due to the compatibility between EP resin and additives, additives are easily separated from the EP resin matrix when the composite is used for a long time, resulting in the mechanical properties of the EP resin composite [10,11,12]; therefore, the development of efficient, inherently flame-retardant epoxy resin products with multi-ring structures has become a research focus.

Recently, some fillers available in the commercial market, such as carbon nanotubes [13], multiwalled carbon nanotubes [14], functionalized graphene [15,16] and hexachlorocyclotriphosphazene (HCCP) [17,18], have been used to improve the flame retardancy of epoxy resin, and excellent results have been obtained. Qu [16] synthesized reactive cyclophosphazene and grafted it onto graphene oxide to prepare flame-retardant graphene oxide (fGO) nano sheets for EP nanocomposites. The obtained fGO showed good compatibility and improved the thermal stability and dynamic mechanical properties of the composite. Among the fillers mentioned above, HCCP is a classic example of reinforcing elements for polymeric composites because of its high reactivity, low cost and nearly unlimited modifiability. More importantly, HCCP can improve the flame retardancy of epoxy resin by introducing flame-retardant groups while building a multi-ring structure to improve the heat resistance.

In this investigation, a novel HCCP derivative with a multiple-ring structure (named HCCP-SA) was successfully synthesized though bonding SA into the conjugated ring of HCCP. Then, HCCP-SA was embedded into the main chain of epoxy resin by the ring-opening curing reaction of epoxy resin to prepare a heat-resistant and flame-retardant EP composite (named E-HS-*x*: *x* denotes the parts of HCCP-SA used in per hundred parts of EP resins). The effects of HCCP-SA on thermal property and flame retardancy in the E-HS-*x* composite are discussed in detail.

## 2. Materials and Methods

### 2.1. Materials

HCCP (98%) and epoxy resin (E-51) were provided by Sigma-Aldrich Co. Ltd. (Shanghai, China); salicylamide (SA) was provided by Shanghai Tengzhun Biotechnology Co. Ltd. (Shanghai, China); n-heptane (A.R.), triethylamine (A.R.), diethylenetriamine (A.R.) and tetrahydrofuran (A.R.) were purchased from Changzhou Tongcheng Co., Ltd. (Changzhou, China).

### 2.2. Synthesis of HCCP-SA

**Figure 1:**
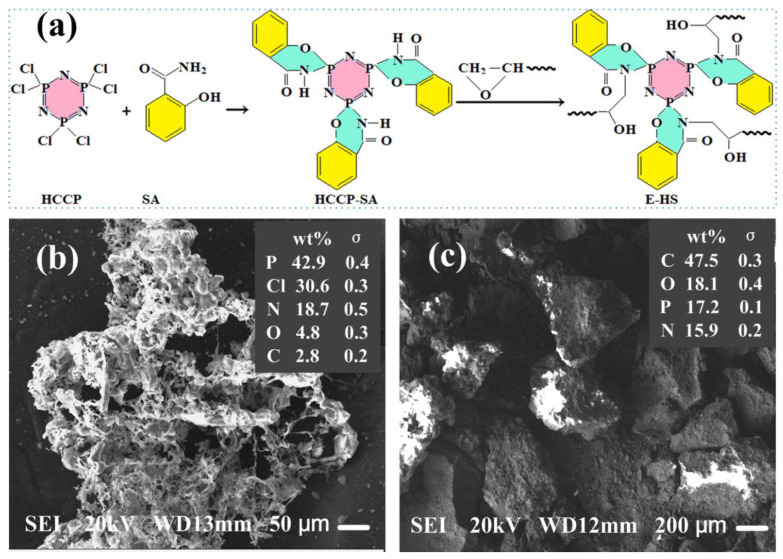
Synthesis route (**a**) and SEM (**b,c**) of HCCP and HCCP-SA; the inset shows the element content measurement result by EDS.

HCCP (13.90 g, 0.04 mol) and tetrahydrofuran (120 mL) were placed in a three necked flask and stirred vigorously for 0.5 h at 65 °C in a nitrogen atmosphere. SA (41.90 mL) and triethylamine (10.00 mL) were mixed evenly and added drop by drop into the three necked flask with a constant pressure-dropping funnel within 20 min. The reaction was kept at 65 °C for approximately 8 h under a nitrogen atmosphere. Then, vacuum distillation was performed on a rotary evaporator, model RE520 (Yarong, Shanghai, China), at 80 °C, to remove the solvent (tetrahydrofuran). The crude product was washed with n-heptane and deionized water 3 times, and then dried at 60 °C for 36 h in a vacuum oven. Finally, white powder HCCP-SA was obtained after grinding. Figure 1a shows the synthesis route of HCCP-SA and its multiple-ring structure.

### 2.3. Preparation of E-HS-x Composites

According to the calculated mass, appropriate amounts of diethylenetriamine were added into a beaker containing EP resin and stirred quickly for 3 min at room temperature. Immediately after, HCCP-SA was added into the above precuring epoxy resin and stirred quickly for 3 min. The mixture was injected into the molds with a cavity size of 100 mm × 10 mm × 4 mm (comprising polytetrafluoroethylene) as soon as possible to prepare the E-HS-*x* composite. Detailed experimental procedures for the preparation of the E-HS-*x* composite are reported in our previous investigations [18]. The mass of HCCP-SA used in the preparation of E-HS-*x* composite is listed in Table 1, in which phr is defined as the dosage per hundred grams of EP.

### 2.4. Characterization

Infrared measurements were recorded on a Nicolet IS10 Fourier transform infrared (FTIR) spectrometer (ThermoFisher, Waltham, MA, USA) to obtain the FTIR spectra of the SA, HCCP-SA and E-HS-*x* composites. The ^1^H NMR (400 MHz) spectra of HCCP-SA were recorded on a AVANCE III 400M nuclear magnetic resonance spectrometer (NMR, Bruker, Romanshorn, Switzerland), using Hexadeuterated dimethyl sulfoxide (DMSO-d6) as the solvent. The morphology of the E-HS-*x* composites was recorded using a scanning electron microscope (SEM, Sigma 500, Carl Zeiss, Germany). The thermogravimetric analysis (TGA) data for the E-HS-*x* composites were investigated separately by a TA Instruments DTA7300 (Waltham, MA, USA) at a heating rate of 20 °C min^−1^, from 20 °C to 700 °C, under a N_2_ atmosphere. The limiting oxygen index (LOI) values of the E-HS-*x* samples were characterized by using an oxygen index meter (JF-3, Jiangning, China), according to GB/T2406.2-2009. The Raman spectra and X-ray photoelectron spectroscopy (XPS) of the char residues were measured by spectrometer models, Dxr2xi and ESCALAB 250Xi (ThermoFisher, Waltham, MA, USA), respectively. The combustion properties of the EP and E-HS-*x* composites were investigated by a cone calorimeter (CC), iCone Classic (FTT., East Grinstead, UK), according to ISO-5660-1 standard. The thermal radiation power was 50 kW/m^2^ in CC testing, and the size of the sample was 100 mm × 100 mm × 4 mm.

## 3. Results and Discussion

### 3.1. Morphologies and Element Analysis of HCCP-SA

The morphology of HCCP and HCCP-SA was recorded by SEM and is summarized in Figure 1b,c, respectively; furthermore, the insets of Figure 1b,c show the element content measurement result of HCCP and HCCP-SA by EDS. It can clearly be seen from Figure 1b that the N, Cl and P element amounts of HCCP were 18.7%, 30.6% and 42.9%, respectively, which are close to the theoretical values of those of HCCP. When the nucleophilic substitution reaction of SA and HCCP was completed, the amount of N and P in the HCCP-SA molecule was 17.2% and 15.9%, respectively, as shown in Figure 1c.

It can be clearly observed from Figure 1b that HCCP presented an irregular loose network structure with many significant large pores, which can increase the specific surface area of HCCP and enable more functional groups to appear on the surface, thus, speeding up the nucleophilic substitution reaction between HCCP and SA. As shown in Figure 1c, the reactant HCCP-SA exhibited an irregular massive structure with a diameter of about 300 μm. In the process of the nucleophilic substitution reaction, the -NH_2_ and -OH groups in the SA molecule lost H atoms and were bonded to the conjugated aromatic ring of HCCP through the N-P and O-P bond [19], respectively, and finally formed a larger HCCP-SA block.

### 3.2. Structural Characterization of HCCP-SA

**Figure 2:**
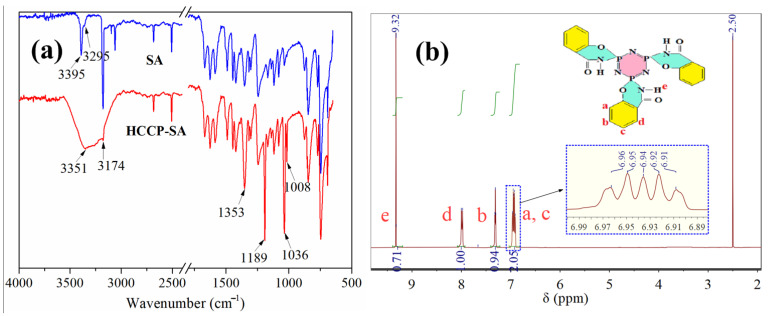
FTIR (**a**) and ^1^H NMR (**b**) of SA and HCCP-SA. In (**b**), a, b, c, d and e represents the peak of H atom at a, b, c, d and e in the molecular formula of HCCP-SA, respectively.

Figure 2a shows the FTIR spectra of the HCCP-SA and SA specimens. In the SA spectrum, the -CONH- motion peak centered at 3170 cm^−1^, and the primary amino peaks centered at 3395 cm^−1^ and 3295 cm^−1^ are easily observed. The stretching vibration absorption peaks of the C=C bond in the aromatic rings appeared from 1450 cm^−1^ to 1600 cm^−1^. Two vibrational absorption peaks appeared at 1280 cm^−1^ and 1353 cm^−1^ and were assigned to C-N and C-O, respectively [16]. For HCCP-SA, the peaks near 1189 cm^−1^ were assigned to the motions of P=N. Two new vibrational absorption peaks appeared at 1008 cm^−1^ and 1036 cm^−1^, which were assigned to P-N and P-O, respectively. Wide bands at 3351 cm^−1^ were assigned to the secondary amino. The FTIR results, especially the appearance of the absorption peaks of three new chemical bonds (i.e., P-O, P-N and secondary amino -NH-), primarily prove the successful preparation of HCCP-SA [20,21].

An ^1^H NMR analysis was executed to further analyze the structure of the HCCP-SA, and the NMR spectrums are presented in Figure 2b. It can easily be observed in Figure 2b that the chemical shift at 2.50 ppm was apportioned to DMSO-d6, which is used as a solvent for HCCP-SA. The multiple absorption peaks centered at 6.94 ppm (m, 2H) can be attributed to the coupling of H atoms corresponding to H^a^ and H^c^ in the benzene ring structure. The peaks centered at 7.31 (t, *J* = 6.6, 1H) and 7.98 ppm (d, *J* = 2.8, 1H) can be attributed to H^b^ and H^d^ in the benzene ring structure, respectively. A single strong peak at 9.32 ppm (s, 1H) was assigned to H^e^ atoms, which was the only secondary amino hydrogen atom (i.e., -NH-) in the molecule HCCP-SA [22,23]. As evidenced by the FTIR analyses in the previous section, the results of the ^1^H NMR analysis further prove that HCCP-SA was successfully prepared.

### 3.3. Thermal Stability of EP and E-HS-x Composites

**Figure 3:**
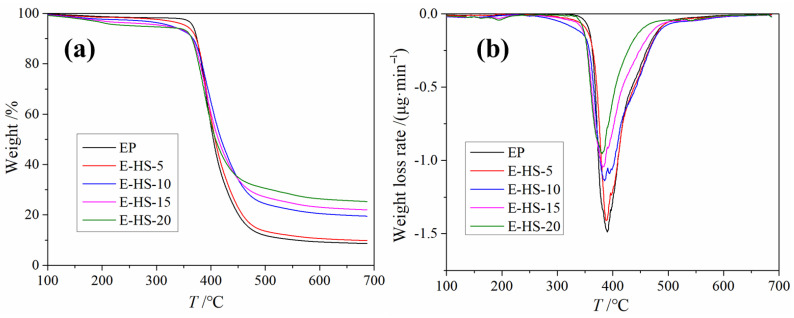
TG curves (**a**) and weight loss rate (**b**) of EP and E-HS-x.

In order to understand the thermal stability of the EP and E-HS-*x* composites, TG curves were tested, and these are summarized in Figure 3a. Under the nitrogen (N_2_) atmosphere, EP resins without flame retardant showed the initial decomposition temperature (*T*_5 wt.%_) and maximum decomposition temperature (*T_max_*) at 367.07 and 390.04 °C, and the char yield of the EP was 8.64% at 700 °C, respectively, which was mainly ascribed to the degradation reaction of the EP molecular chain. Compared with the pure EP resin, the char yield increased from 9.79% to 25.30% at ~700 °C, but the *T*_5 wt.%_ of the E-HS-*x* composites reduced from 355.20 °C to 267.94 °C, respectively, when the amount of HCCP-SA increased from 5 to 20 phr. Moreover, as shown in Figure 3b, the *T_max_* of the E-HS-*x* composites reduced from 387.43 °C to 382.21 °C, and the weight loss speed of the EP and E-HS-*x* composites reduced from 1.49 to 0.95 mg·min^−1^. This indicates that the added HCCP-SA can decompose and absorb the energy of the system at a lower temperature, reducing the decomposition rate and *T_max_* of the E-HS-*x* composites, and thus, preventing further degradation of the EP resin matrix. In a word, the multiple-ring cross-linking network structure composed of the benzene ring, P-N, and N-O heterocycles is the main reason why E-HS-*x* composites have outstanding heat resistance [24].

### 3.4. Flame Retardancy of EP and E-HS-x Composites

LOI and UL-94 tests were carried out in order to understand the flame retardancy of the EP and E-HS-*x* composites. In the UL-94 test, the E-HS-*x* composites were made into splines in the size of 125 mm × 13 mm × 13 mm, according to ASTMD635. Table 1 presents the composition of the EP and E-HS-*x* composites and the determination results of LOI and UL-94 testing, in which the amount of diethylenetriamine (used as a curing agent) in EP was higher than that in E-HS-*x*. The reason for this is that the -NH- and -OH groups in HCCP-SA participated in the ring-opening curing reaction of the epoxy resin, acting as a curing agent, which is verified by FTIR in Section 3.2. The LOI value of EP resins without flame retardant was 22.4%, and no rating was measured in UL-94 testing.

It is worth noting that the LOI value of the E-HS-*x* composite increased from 24.1% to 25.3%, but no rating was measured in the UL-94 tests, as the amount of HCCP-SA increased from 5 to 15 phr. The reason for this is that the “blowing-out effect” of the N element and the synergistic flame-retardant effect of N-P were not obvious when the content of the P and N element was small, which is insufficient to significantly improve the flame retardancy of the E-HS-*x* composites. Encouragingly, as expected, the E-HS-*x* composites showed excellent flame retardancy when the additional amount of HCCP-SA reached 20 phr. The LOI value of the E-HS-20 composite was as high as 27.1%, which is 20.9% higher than that of pure EP. Meanwhile, there was no dripping in UL-94 testing, even reaching the V-2 rating. This is because the EP molecular chain was entangled with the branch chain on the HCCP-SA conjugate ring to form a network structure, which increased the viscosity of the EP composites and prevented the generation of droplets. In addition, the thickness and density of the surface protective layer of the E-HS-*x* composites were increased by the functional groups (P=O, P-O and P-N) generated by the ring-opening curing reaction, which could effectively prevent heat and O_2_ from contacting the internal substrate of the epoxy resin.

**Figure 4:**
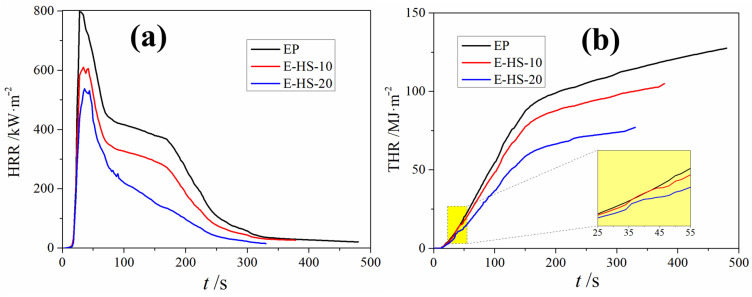
HRR curves (**a**) and THR curves (**b**) for samples under an external heat flux of 50 kW/m^2^.

Cone calorimetry tests were implemented to simulate the combustion behavior of a real fire hazard. In order to achieve efficient measurement and comparability, EP, E-HS-10 and E-HS-20 were selected to measure the following six main indicators: HRR (peak heat release rate), THR (total heat release), T-HRR (time to peak heat release rate), total smoke release, average CO and CO_2_ yield. The results are shown in Figure 4 and Table 2. EP without flame retardant burns violently and releases a large amount of heat immediately after ignition, reaching the HRR of 798 kW/m^2^ in just 28 s (see Table 2 and Figure 4a). However, the HRR value of E-HS-10 and E-HS-20 was 609 (taking 34 s) and 537 (taking 36 s) kW/m^2^, respectively, which indicate that the combustion process is delayed by HCCP-SA linked to the EP molecular chain. It can be clearly observed from Figure 4b that the THR of the EP, E-HS-10 and E-HS-10composites was 127 MJ/m^2^, 105 MJ/m^2^ and 77 MJ/m^2^, respectively, which indicates that HCCP-SA can terminate the combustion process of E-HS-*x* in advance. The E-HS-20 composites presented a distinctly reduced HRR (32.7.0%) and THR (39.4%) compared with those of the pure EP. Furthermore, total smoke release (50%), average CO yield (46.8%) and average CO_2_ yield (31.3%) both gradually reduced with an increasing amount of HCCP-SA (see Table 2). Thus, the cone calorimeter results confirmed the beneficial effect of the HCCP-SA in boosting the flame-retardant performance of the E-HS-*x* composites.

In order to estimate the fire hazard of the composites, the fire growth rate (FGR) was defined and calculated by the following equation:

(1) $$\mathrm{FGR}=\frac{\mathrm{HRR}}{\mathrm{T-HRR}}$$

A large number of studies have proved that the lower the FGR value, the higher the fire safety of the corresponding material [25,26,27]. The FGR values of the EP, E-HS-10 and E-HS-20 specimens were calculated, and these are listed in Table 2. As expected, the FGR value showed a significant downward trend, reducing from 28.5 kW·m^−2^·s^−1^ (EP) to 14.9 kW·m^−2^·s^−1^ (E-HS-20), which significantly demonstrated that the fire safety and flame retardancy of EP could be significantly improved by HCCP-SA after being embedded into the EP molecular chain through a ring-opening curing reaction.

### 3.5. Char Residue Analysis

#### 3.5.1. Morphologies Analysis

**Figure 5:**
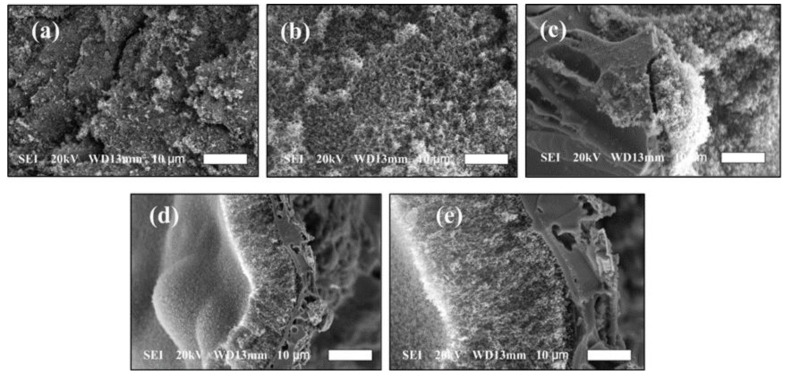
SEM of char residues of: (**a**) EP, (**b**) E-HS-5, (**c**) E-HS-10, (**d**) E-HS-15 and (**e**) E-HS-20.

Figure 5a–e illustrates the SEM of the char residues (CRE) acquired by the EP and E-HS-*x* composites. Figure 5a shows that the CRE of EP without flame retardant was finer and more uniform, which could theoretically prevent EP from continuing to burn. However, the CRE on the surface of EP was not dense enough, even accompanied by many cracks, which means that most of the combustible gases could pass through the cracks during the combustion process and reach the surface of the EP to intensify the combustion. At the same time, the heat could be quickly released to the interior of EP, so that the EP matrix could be rapidly decomposed by heat.

**Figure 6:**
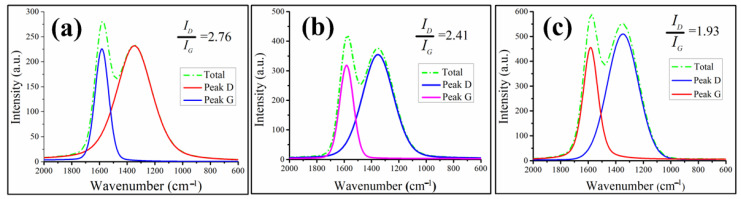
Raman spectra of char residues: (**a**) EP, (**b**) E-HS-10 and (**c**) E-HS-20.

Encouragingly, it is evident in Figure 5b–e that EP added with HCCP-SA formed a dense CRE protective layer on the surface of the E-HS-*x* composite after combustion. Moreover, the thickness of CRE increased with the increase in HCCP-SA addition. H_4_P_2_O_7_ and H_3_PO_4_, with a powerful dehydration effect during combustion, were produced by the presence of abundant N and P containing functional groups in HCCP-SA. In particular, a great deal of CRE was obtained after combustion, which interfered with the thermal degradation process of the EP composites, as well as improved their thermal stability. The above results are confirmed by the Raman spectroscopy analysis of CRE, which is demonstrated in Figure 6.

#### 3.5.2. Raman Spectroscopy Analysis

Raman spectroscopy analysis is a powerful method that can characterize the graphitization degree of CREs acquired by the combustion of organic macromolecular composites. A higher degree of graphitization indicates a better effect of the char layer. Let *I_D_/I_G_* (the integral area ratio of peak D and G) represent the graphitization degree of the char layer. The smaller *I_D_/I_G_* value indicates the stronger organic macromolecular composites’ flame retardancy or ablation resistance, as well as the higher graphitization degree of CREs [28,29]. The Raman spectra of carbon signals typically exhibit a D band at 1360 cm^−1^ and a G band at 1580 cm^−1^, and the D and G bands have different intensities. The calculated *I_D_/I_G_* value of CRE of EP, E-HS-10 and E-HS-20 shown in Figure 6a–c is 2.76, 2.41 and 1.93, respectively, demonstrating that the graphitization degree of CREs, ablation resistance and flame retardancy of E-HS-*x* composites can be effectively improved by HCCP-SA.

#### 3.5.3. XPS Spectra Analysis

**Figure 7:**
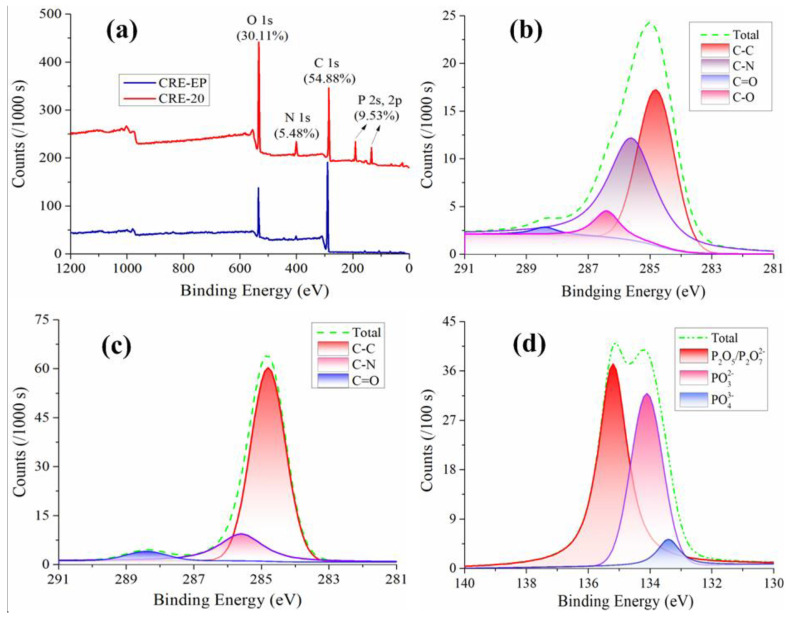
XPS spectra of CREs: (**a**) high-magnification XPS survey spectra of CRE-EP and CRE-20; (**b**) peak-fitting curves of C1s in CRE-EP; (**c**) peak-fitting curves of C1s in CRE-20; (**d**) peak-fitting curves of P2p in CRE-20.

To confirm the bonding form of CREs—which is acquired by the combustion of the EP and E-HS-20 composites (defined as CRE-EP and CRE-20, respectively)—the XPS spectra of CREs were measured, as shown in Figure 7a–d. There were five obvious characteristic peaks at 530.3, 400.8, 284.8, 186.5 and 134.1 eV in the full survey spectra of CRE-20, which correspond to O1s, N1s, C1s, P2s and P2p, respectively, and the tested values are in good agreement with the reported values in references [30,31,32]. In the C1s fitting spectrum of CRE-EP, four kinds of C element bonding forms are as follows: C=O (288.4 eV), C-O (286.4 eV), C-N (285.6 eV) and C-C (284.8 eV) (see Figure 7b) [33,34,35,36]. It is noteworthy that there was no characteristic peak at 286.4 eV in the C1s fitting spectrum of CRE-20 (see Figure 7c). Moreover, the content of carbon oxide in CRE-EP (integral areas of C-O and C=O characteristic peak) was much higher than that in CRE-20 (integral areas of C=O characteristic peak), indicating that it became more difficult for EP to form carbon oxide owing to the addition of HCCP-SA. On the contrary, it could only form a denser and thicker protective carbon layer, which could separate heat, oxygen and the epoxy resin matrix. In Figure 7d, the three kinds of bonding forms of P element in the P2p fitting spectrum of CR-EP and CRE-20 (see) are P_2_O_5_/P_2_O_7_^2−^ (135.2 eV), PO_3_^2−^ (134.1 eV) and PO_4_^3−^ (133.4 eV), respectively [28]. The formation of these phosphides or phosphates absorbs a significant amount of heat, thus, reducing the system temperature and causing the epoxy resin to self-extinguish. These results are consistent with the analysis of FTIR shown in Section 3.2.

#### 3.5.4. FTIR Spectra Analysis

**Figure 8:**
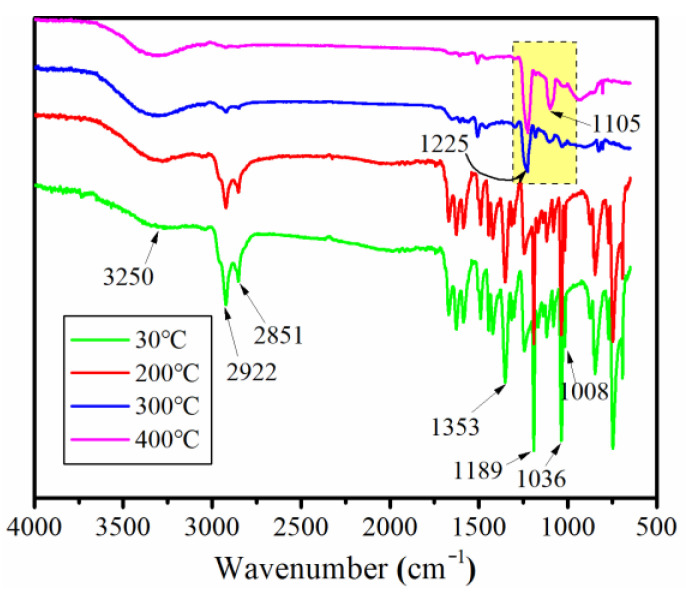
FTIR spectra of thermal decomposition product of E-HS-20 at several representative temperatures.

The FTIR spectra of the thermal decomposition product, obtained by TG at 30 °C, 200 °C, 300 °C and 400 °C, respectively, were utilized to evaluate the thermal degradation process of the E-HS-*x* composites and were illustrated in Figure 8. When the temperature increased from 30 °C to 200 °C, there was no significant change in the intensity of the absorption peak on the two infrared spectral curves. In fact, there was only a small amount of mass loss before 200 °C under an air atmosphere, revealing that E-HS-20 is thermally steady below 200 °C. It is noteworthy that the peaks centered at 2922 cm^−1^, 2851 cm^−1^ and 3250 cm^−1^ belong to the C-H bond and –O-H [15], respectively. This demonstrates that prepared HCCP-SA was involved in the ring-opening curing reaction of epoxy resin. However, the intensity of almost all the absorption peaks decreased significantly, as the temperature was higher than 300 °C, indicating that the decomposition of the E-HS-*x* composites mainly occurred in this phase. Moreover, in the infrared spectral curves corresponding to 300 °C and 400 °C, the appearance of the new peaks centered at 1105 cm^−1^ and 1225 cm^−1^ was allocated to the vibration of the P-O-P and P=O bond, respectively. This indicates that the E-HS-*x* composite generates phosphate, polyphosphate (P_2_O_5_ and P_4_O_10_), H_4_P_2_O_7_ or H_3_PO_4_ with excellent thermal stability after combustion [19,21]. The process of the char layer formation was revealed by the appearances and disappearances of all the characteristic peaks.

### 3.6. Flame-Retardant Mechanism

Based on the above characterization results, we proposed a feasible flame-retardant mechanism of E-HS-*x* composites as follows. Firstly, HCCP-SA decomposes to generate NH_3_ and H_2_O when the temperature is above 267 °C. The vaporization of water absorbs a significant amount of heat and lowers the temperature of the system. Meanwhile, steam and NH_3_ can dilute O_2_ and combustible gas, hence, slowing down the combustion rate of EP composites. Secondly, when the temperature continues to increase, P-containing volatiles in the gas phase can further decompose into PO· and PO_2_· to clear the combustible H· or OH· radicals and achieve a quenching effect. Finally, the char produced by combustion is foamed by steam and NH_3_, resulting in the formation of a compact, thermally stable, and continuous char residue layer, which covers the surface of the EP matrix. The dense char layer with P_2_O_5_, P_2_O_7_^2−^, PO_3_^2−^ and PO_4_^3−^ prevents heat, oxygen and mass loss. The flame-retardant mechanisms were ascribed to barrier effects in the condensed phase and the quenching effect in the gas phase.

## 4. Conclusions

In this study, HCCP-SA with a multiple-ring molecule containing P, O and N elements was successfully synthesized and utilized to prepare EP composites with the dual functions of heat resistance and flame retardancy (i.e., E-HS-*x*). HCCP-SA possessed objective functional groups and a multiple-ring structure (i.e., benzene ring, P-N and N-O heterocycles) through systematic characterizations. Furthermore, the preparation of epoxy resin inherent flame retardants was realized by embedding HCCP-SA in the molecular chain of the resin in the progress of its ring-opening curing reaction.

The E-HS-20 composites presented outstanding heat resistance and flame retardancy. Their initial decomposition temperature (*T*_5 wt.%_) was 267.94 °C and the max weight loss speed was only 0.95 mg·min^−1^, which was reduced by 27.0% and 36.2% than that of pure EP, respectively. In the UL-94 tests, the E-HS-20 composites presented a V-2 rating and LOI at 27.1%, which was increased by 20.9% compared to pure EP. Moreover, the E-HS-20 composites presented a distinctly reduced HRR (32.7%), THR (39.4%), total smoke release (50.0%) and *I_D_/I_G_* value (30%) of CRE-20 compared with those of pure EP. Compact char residue layers and suppressed volatile products indicate that HCCP-SA presents N-P synergistic flame retardancy and a blowing-out effect in the gaseous phase. Meanwhile, the heat, oxygen and mass loss was avoided by the dense char layer, and the flame-retardant mechanisms were due to the barrier effects in the condensed phase, as well as the quenching effect in the gas phase. Therefore, the best-prepared E-HS-*x* composite would be a suitable and potential candidate for heat-resistant and flame-retardant polymer materials, especially electronic packaging and sealing materials.

## Figures and Tables

**Table 1:** Composition of EP and E-HS-*x* and flame-retardancy data.

Specimen	EP/g	Diethylene-Triaminen/phr	HCCP-SA /phr	LOI/%	UL-94	
					Dripping	UL-94 Rating
EP	100	15	0	22.4 ± 0.10	yes	No rating
E-HS-5	100	10	5	24.1 ± 0.22	yes	No rating
E-HS-10	100	10	10	24.3 ± 0.13	no	No rating
E-HS-15	100	10	15	25.3 ± 0.08	no	No rating
E-HS-20	100	10	20	27.1 ± 0.14	no	V-2

**Table 2:** Data from cone calorimetry tests.

Samples	HRR (kW/m^2^)	THR (MJ/m^2^)	T-HRR (s)	Total Smoke Release (m^2^/m^2^)	Average CO Yield (kg/kg)	Average CO_2_ Yield (kg/kg)	FGR (kW/m^2^·s^−1^)
EP	798	127	28	24	0.032	1.6	28.5
E-HS-10	609	105	34	16	0.021	1.2	17.9
E-HS-20	537	77	36	12	0.017	1.1	14.9

## Data Availability

Not applicable.

## List of Notations

**Full Name**	**Notation**
hexachlorocyclotriphosphazene	HCCP
salicylamide	SA
prepared flame retardant (i.e., the product of HCCP and SA reaction)	HCCP-SA
flame-retardant graphene oxide	fGO
epoxy resin	EP
epoxy resin curing products with different quality rates of HCCP-SA	* E-HS-*x*
initial decomposition temperature	*T*_5 wt.%_
maximum decomposition temperature	*T*_max_
peak heat release rate	HRR
time-to-peak heat release rate	T-HRR
total heat release	THR
fire growth rate	FGR
char residues	CRE
char residue acquired by combustion of EP	CRE-EP
char residue acquired by combustion of E-HS-x	* CRE-x
* *x* denotes parts of HCCP-SA used in per hundred parts of EP resins (phr).
